# Clinical effects of *Garcinia kola *in knee osteoarthritis

**DOI:** 10.1186/1749-799X-3-34

**Published:** 2008-07-30

**Authors:** Olayinka O Adegbehingbe, Saburi A Adesanya, Thomas O Idowu, Oluwakemi C Okimi, Oyesiku A Oyelami, Ezekiel O Iwalewa

**Affiliations:** 1 Department of Orthopaedic Surgery and Traumatology, Faculty of Clinical Sciences, Obafemi Awolowo University, Ile-Ife, Nigeria; 2 Professor of Pharmacognosy, Department of Pharmacognosy, Faculty of Pharmacy, Obafemi Awolowo University, Ile-Ife, Nigeria; 3 Department of Pharmaceutical Chemistry, Faculty of Pharmacy, Obafemi Awolowo University, Ile-Ife, Nigeria; 4 Department of Nursing Sciences, Faculty of Basic Sciences, Obafemi Awolowo University, Ile-Ife, Nigeria; 5 Professor of Pediatrics and Child Health, Department of Pediatrics and Child Health, Faculty of Clinical Sciences, Obafemi Awolowo University, Ile-Ife, Nigeria; 6 Department of Pharmacology, Faculty of Pharmacy, Obafemi Awolowo University, Ile-Ife, Nigeria

## Abstract

**Objectives:**

Over the past years, there has been a growing number of knee osteoarthritis (KOA) patients who are not willing to comply with long-term non-steroidal anti-inflammatory drugs (NSAID) treatment and wish to use herbal anti- rheumatic medicine. This study assessed the clinical effects of *Garcinia kola *(GK) in KOA patients.

**Patients and methods:**

Prospective randomized, placebo controlled, double blind, clinical trial approved by the institutional medical ethics review board and written informed consent obtained from each patient. All KOA patients presenting at the Obafemi Awolowo University Teaching Hospital complex were recruited into the study. The patients were grouped into four (A = Placebo, B = Naproxen, C = *Garcinia kola*, D = Celebrex). The drugs and placebo were given twice a day per oral route. Each dose consisted of 200 mg of *G. kola*, Naproxen (500 mg), Celebrex (200 mg) and Ascorbic acid (100 mg). The primary outcome measure over six weeks study period was the change in mean WOMAC pain visual analogue scales (VAS). Secondary outcome measures included the mean change in joint stiffness and physical function (mobility/walking).

**Results:**

143 patients were recruited, 84 (58.7%, males – 24, females – 60) satisfied the selection criteria and completed the study. The effect of knee osteoarthritis bilateralism among the subjects was not significant on their outcome (p > 0.05). The change in the mean WOMAC pain VAS after six weeks of *G. kola *was significantly reduced compared to the placebo (p < 0.001). Multiple comparisons of the mean VAS pain change of *G. kola *group was not lowered significantly against the naproxen and celebrex groups (p > 0.05). The onset of *G. kola *symptomatic pain relief was faster than the placebo (p < 0.001). However, it was slower than the active comparators (p > 0.05). The duration of therapeutic effect of *Garcinia kola *was longer than the placebo (p > 0.001). *G. kola *period of effect was less than naproxen and celebrex (p < 0.001). *G. kola *subjects had improved mean change mobility/walking after six weeks better than the control group(p < 0.001). The mean change in mobility of the *G. kola *group when compared to the active comparators was not significantly better (p < 0.05). The mean change of knee joint stiffness (p < 0.001) and the change of mean WOMAC score (p < 0.001) were improved on *Garcinia kola *as compared to the placebo. The mid term outcome of eleven *Garcinia kola *subjects after cessation of use had a mean pain relief period of 17.27 +/- 5.15 days (range: 9–26 days). There was no significant cardiovascular, renal or drug induced adverse reaction to *Garcinia kola*.

**Conclusion:**

*Garcinia kola *appeared to have clinically significant analgesic/anti-inflammatory effects in knee osteoarthritis patients. *Garcinia kola *is a potential osteoarthritis disease activity modifier with good mid term outcome. Further studies are required for standardization of dosages and to determine long-term effects.

## Background

Osteoarthritis is the most common form of joint disease, affecting the knee more than other joints [1]. Several factors play a role in osteoarthritis risk; these include age, gender, genetics, behavioral influences and ethnicity [2]. Trauma is a recognized predisposing factor to development of osteoarthritis of the knee (KOA) associated with raised intra osseous pressure and death of the chondrocytes. Osteoarthritis of the knees reduces the ability to avoid obstacles and supporting epidemiologic studies have found osteoarthritis to be a risk factor for falls [3]. The pain associated with osteoarthritis of the knees increased the propensity to trip on an obstacle and underscores the importance of treating pain associated with osteoarthritis [3].

As the population ages or the disease worsens, knee osteoarthritis is associated with incapacity and a deteriorating quality of life owing to increased pain, loss of mobility, and the consequent loss of functional independence [4]. There is general increase in life expectancy with increasing involvement of the younger age group in foot ball, road traffic injuries, political/communal wars and disaster. It means that increasing numbers of people will present with reduced quality of life associated knee osteoarthritis. As a result, osteoarthritis is often treated by medical or surgical intervention. Pain relief is therefore a fundamental aspect in dealing with this illness.

In patients in whom pharmacological treatment is ineffective, who are not candidates for surgery (or who reject it); other pain and predisposing factors management procedures should be considered [5]. Drug therapy of osteoarthritis is empirical and largely directed towards providing symptomatic relief, primarily by the use of analgesics and non-steroidal anti-inflammatory drugs (NSAIDs). Over the past years, there are a growing number of younger age group patients with KOA patients who are not willing to comply with long term NSAIDs treatment and who wish to use more naturally occurring ant rheumatic medicine.

*Garcinia kola *Heckel of the family Guttiferaceae [6] is called Kola bitter, Bitter Kola, False or Male Kola. The Nigerian names are Adu, Ugolu in Ibo language; Orogbo in Yoruba; and Akan in Tweapia language. The constituents include- Flavinoids (bioflavonoid), xanthenes and benzophenones. It has shown anti-inflammatory, ant parasitic, antimicrobial and antiviral properties [[7-12], and [13]].

The *pharmacodynamic mechanism *of *Garcinia kola *action is anchored on Kolaviron (KV) [[14-17], and [18]]. *Garcinia kola *acts by restoring and maintaining the balance of fatty acids in osteoarthritis. It causes balanced inhibition of metabolism in the cyclo oxgenase pathway (COX-1 and COX-2). It inhibits amino acid metabolism by the 5-lipoxygenase (5-LOX) enzymes. Therefore the normal physiologic organ functions are maintained. The inhibition of 5-LOX result into reduction in the production of leukotrienes (LTB4), an agent that do enhance white blood cell chemo taxis and the subsequent release of histamines, reactive oxygen species and pro-inflammatory cytokines. *Garcinia kola *has a strong antioxidant effect which limits the oxidative conversion of amino acid by reactive oxygen species to other damaging fatty acid products [19]. Kolaviron exerts a hypocholesterolaemic effect which illustrate the anti-atherogenic property of *G. kola *[20]. The excretion has neither organ nor behavioral abnormalities. Blood electrolytes were unchanged and liver enzymes and markers of renal function were all within normal limits [14,16].

### Safety of the food effects

*Garcinia kola *is safe taken with or without other foods. Taking it an hour before or after meals may help to increase the absorption of the key ingredients. Food does not affect the metabolism of *G. kola *and may buffer effects of mild indigestion [17]. *Garcinia kola *is primarily carried bound to albumin in the blood and only a minor amount is metabolized by hepatic metabolism [18]. Kolaviron does not affect phase 1 drug metabolizing enzymes [16] but induce phase 2 enzymes [21]. It may be classified as a bifunctional inducer according to the classification of Greenwald et al 1995 [22].

*Drug interactions *of Kolaviron have been shown to be hepatoprotective [[7,10,21,23,24], and [25]]. It does not appear to have a pronounced effect on drug metabolizing enzymes [21] and no known interactions with orthodox medications [26]. The tablet properties could be controlled to obtain optimal release of the bioactive compounds [27].

Although surgery can relieve the pain of KOA and restore function, not all patients are candidates for surgery, and many want to avoid or delay it if possible. Therefore, alternative treatments are important [28] which could include *G. kola *that have not been evaluated for knee osteoarthritis. To date, there is no clinical documentation of the effect of *G. kola *on the knee osteoarthritis through a Medline search and locally available literatures. The research hypothesis was that *G. kola *have a positive effect on knee osteoarthritis as depicted in Yoruba folk songs. The objective of the study was to evaluate the clinical effects of G kola on knee osteoarthritis pain, stiffness and function. The effects of *G. kola *in KOA is been investigated in Nigerians through a multidisciplinary research study group based at the Obafemi Awolowo University, Ile-Ife, Osun State; Nigeria.

## Methods

### Inclusion criteria

Men and women between the ages of 18 and 80 years were enlisted if they had knee trauma or overuse of the knees prior to the onset of symptomatic osteoarthritis. Only uncomplicated hypertensive patients solely on Nifedipine were included. Within the routine clinical practice, many of our confirmed KOA patients do present with hypertensive heart disease on multiple therapies. Basically our research center is in resource constrained country where high technology laboratory support needed to identify specific adverse drug interactions that could be associated with multiple anti hypertensive medications is not available. It was due to this peculiar limitation and the need to enhance clarity of interpretation of subject's clinical features, study drug's efficacy and safety assessment within available international standard facilities that necessitated the inclusion of patients with uncomplicated hypertensive solely on Nifedipine.

Patient's diets were not altered from their pre study period. The other inclusion criteria in the study were – osteoarthritis of the knee verified according to the clinical, laboratory, radiographic criteria of the American College of Rheumatology (ACR) and resting visual analog scale pain intensity in the target knee > 45 mm.

### Exclusion criteria

Patients with known allergic reactions (to *Garcinia kola*, celebrex and naproxen); Kidney failure (blood creatinine > 84 umol/L); abnormal liver function (SGOT > 35 U/L or SGPT > 35 U/L or GGT > 50 U/L); gastrointestinal ulcers (bleeding or discolored stool during the past 8 weeks); malignant diseases; systemic therapy with corticosteroids during the past 8 weeks; surgery of the test joint during the past 8 weeks; inflammatory joint diseases(ESR > 40 mm/h);chronic heart failure (NYHA grade III or IV); Chronic obstructive airway disease requiring prophylactic medication and participation on a clinical trial during the past 4 weeks. Patients with absolute indication for surgery and knee instability were excluded. Subjects with high levels of clinical disability (> 34 on the WOMAC scale) were excluded as it was unlikely that they could perform the experimental protocol consistently. Also the knee requiring no weight bearing and who had intra-articular injections into the study joint within 3 months prior to first visit were excluded. No herbal or allopathic treatment, which could influence the outcome of the study, was permitted during the course of the study. Crossing over of patients from one subgroup to another was excluded through surveillance.

### Settings

Subjects were recruited into the study between February 20^th^, 2004 and August 19^th^, 2006 at the orthopedics and general outpatient's clinics of the Obafemi Awolowo University Teaching Hospital Complex, Ile-Ife (OAUTHC); Nigeria.

### Study medication and blinding

At the beginning of the study, no commercial preparation of *Garcinia kola *was available. *G. kola *seeds were obtained from a market in Ile-Ife, authenticated by Mr. A.T. Oladele, the herbarium, Department of Pharmacognosy, Obafemi Awolowo University.

*G. kola *200 mg was given twice orally per day for six weeks. Active comparators were Naproxen 500 mg tablets twice daily, and Celebrex 200 mg twice daily were given orally. Placebo study medication was ascorbic acid, 100 mg tablet given twice daily. The patient in each group was given identical study medication of the same physical appearance aimed at eliminating psychological effects on the subjects. The subjects have not been previously exposed to research drug medications.

All study medications were prepared by a 10 years post-qualified nursing staff and administered to each patient by a senior registrar in orthopedic surgery. A family medicine physician of five year post fellowship qualification acted as a masked clinical outcome evaluator. The voluntary nursing staff, the senior registrar and masked evaluator were not part of the study group. The Nifedipine for hypertensive were supplied to patients from the teaching hospital pharmacy shop to ensure uniformity of quality control. A consultant radiologist with a neutral role status in the study screened the plain X-ray of patients and confirmed radiological features of knee osteoarthritis.

### Study design

It was a randomized, double-blind, placebo-controlled, parallel-group study trial of the clinical effects of *G. kola *in knee osteoarthritis. The research medication dose assessor, clinical assessors, subjects and orthopedic surgeons were blinded to the treatment group for the six weeks of study. The *G. kola *subgroup was followed up for the midterm evaluation of therapeutic effect after the intake was discontinued.

The study was approved by the institutional medical ethics review board and was carried out in accordance with the ethical principles of the Declaration of Helsinki. A written informed consent was obtained from each patient that fulfilled the inclusion criteria before randomization.

### Study randomization

To determine the presence of KOA, detailed history was obtained and clinical examination performed. Adults were classified as having clinical knee OA by using the criteria determined by the American College of Rheumatology (ACR) [29].

Randomization occurred in blocks of four within each stratum, using computer generated random numbers (Excel 5.0). The patients were grouped into four (A = placebo, B = naproxen, C = *Garcinia kola*, D = celebrex). Both assessors and patients were blind to the allocation and not informed about the block size until after completion of the study. Subjects were treated for six weeks and each of them had weekly follow-up visits until the time of study withdrawal. Study medications were taken in the morning and at noon, half an hour before meal times. No additional analgesics, NSAIDs or systemic corticosteroids were allowed during the study phases.

### Objectives

At all visits, the patients completed WOMAC index to assess pain, stiffness, and physical function. The primary efficacy end point was the mean change from baseline in the WOMAC pain subscale VAS at six weeks. Secondary outcome measures included the stiffness and physical function.

### Clinical assessments

Patients were assessed by consultant orthopedic surgeon at days -7, 0, 7, 14, 21, 28, 35 and 42.

The *initial assessment (day -7) *comprised of medical history, examination and WOMAC-VAS index. The "most painful knee joint" refer to the side of knee joint with the highest VAS pain score when a subject is having bilateral KOA. In bilateral knee OA, the side of the knee joint with the highest VAS pain score was the primary focus of measurement for future assessment in the study. Blood and urine samples were taken for standard laboratory tests. The onset of therapeutic effect of the study medication as used in this study was the mean time (minutes) recorded for the onset of KOA symptomatic reliefs. The duration of therapeutic effect of the study medication was the mean time (minutes) recorded for the return of KOA symptoms after a relief following study medication intake.

The *radio diagnostic criterion of Kellgren and Lawrence *[30]*scheme *was used for the knee osteoarthritis severity assessment. All patients underwent radiographic analysis of both knee joint using Kellgren-Lawrence less or equal to grade 2 as case definition.

The level of *clinical disability *was quantified by using the Western Ontario and McMaster University Osteoarthritis (WOMAC) index [30]. Disability was assessed using the physical function section of WOMAC, which contains 17 questions relating to functional disability, scored from 0 to 4 by the subject.

At the *second study visits (day 0)*, laboratory findings were compared with exclusion criteria and diary entries were checked. Patients who met all study criteria filled in the WOMAC questionnaire (baseline), received study medication and thereby entered the intent to treat population.

At the *study visits on days 7,14,28,35, and 42*, patients filled in WOMAC questionnaires, and compliance was checked by diary entries and study medication. Adverse events were recorded by checking diary entries as well as by direct questioning of the patients.

At the *study visit on day 42*, blood and urine samples were taken. The masked clinical outcome evaluator and consultant orthopedic surgeon independently recorded their final overall assessment of the change of disease activity by the study medication on 100 mm visual analogue scales (VAS). Direct inquiry and visual inspection of the returned sachets container were used for monitoring compliance. The osteoarthritis protocol instrument used for the study has a section on the disease symptom/symptom description. It evaluates each subject from the day 0 to the end of study at six weeks. The subjective response of the patients to the questions on walking distance was distinct. They were asked if the distance used to cover by walking has improved, or there is no change, or it deteriorated at the end of the study compared to the day 0.

### Safety

The subjects who had received at least a dose of the research medication s were assessed for their safety. Tolerability evaluations consisted of determining clinical laboratory test abnormalities such as hepatic (aminotransferase activities) and renal (serum creatinine) function, adverse events, and physical examinations. Adverse events reported by the patient or observed by the investigator during clinical evaluation were recorded. In addition, patients were questioned at each visit regarding the occurrence of adverse events using a nonspecific question. Investigators rated the intensity of adverse events and their subjective assessment of the relationship to study medication while blinded to the treatment group.

### Statistical analyses

All analyses were performed on the intention to treat cohort, defined as all patients who took at least a dose of study medications. Data were analyzed by using Statistic Package for Social Sciences (SPSS) version 11.0 for windows. The comparability of patients in the four treatment groups was determined from the demographic data and baseline haemodynamic values. The change in the mean of the group and mean time of study medications therapeutic duration were evaluated using 2-way ANOVA with Post Hoc comparison test. The confidence interval (CI) was at 95% and the P-value was considered significant at p ≤ 0.05.

### Study limitations

Limited numbers of knee osteoarthritis patients were available for the study. Lack of research grants to provide study medications and laboratory investigations free for long term and to include larger groups of would be subjects at multiple centers in Nigeria.

## Results

### Demographic characteristics

**Figure 1:**
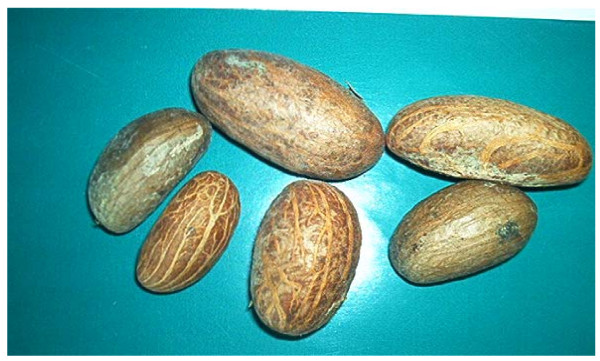
*Garcinia kola *seeds of various sizes.

A total of 143 patients who had post traumatic knee osteoarthritis (unilateral = 94, bilateral = 98) on 192 limbs were recruited. Eighty-four patients (58.7%) with KOA in 121 limbs satisfied the selection criteria. All the patients who received at least a dose of the research drugs had adequate documentation of their data in each subgroup and were used for analysis. There were 24 males (28.6%) and 60 females (71.4%) with M: F: 1:2.5. The proportion of bilateral knees involvement is shown in Table 1 which compared patients in the four treatment groups. The effect of knee osteoarthritis bilateralism among the subjects was not significant (p > 0.913). The WOMAC score at day 0 was not significantly different among the four study groups (p > 0.05). A clinical photographs of typical *Garcinia kola *is illustrated in Figure 1. The major sources of trauma were Road Traffic Accident 46(54.8%), Sports injury 19(22.6%), Fall from height 18 (21.4%) and prolonged over use from driving long distance for over 25 years, 1(1.2%). Knee osteoarthritis in young adults was common after sporting knee injury and fall from height. The women often were those carried as passenger on motor cycle before they sustained injury. The mean duration of trauma before the onset of symptomatic knee osteoarthritis for males was 17.4 years +/- 7.3 and females 14.2 years +/- 8.6. There was 100% compliance rate in the control and celebrex groups. A patient (4.76%) in the naproxen group was unable to come to the hospital for the last two days evaluation due to diarrhea. He was traced home for evaluation and discovered the medication was taken. Two patients in the *Garcinia kola *group stopped after completion of 39 and 40 days on the medication. It was due to light headedness and palpitation in each of the patients. Both patients were hypertensive before the commencement of the study. They were lost during the mid term follow up period of the study. The data from the patients were accepted for analysis after completion of 92.8% and 95.2% of the six weeks study period.

**Table 1:** Baseline demographic and clinical characteristics of all randomized patients.

**Demographic** ** characteristics**	**TRT GR A**	**TRT GR B**	**TRT GR C**	**TRT GR D**
Mean age(SD)	53.2(6.0)	51.0(7.3)	54.1(5.8)	52.5(7.1)
Age range(yr)	36–61	34–59	34–60	35–62
Sex				
Female	14	15	15	16
Male	7	6	6	5
Race (%Black)	100.0	100.0	100.0	100.0
Ethnic origin(% Yoruba)	100.0	100.0	100.0	100.0
Religion(%Christianity)	80.9	76.2	90.4	80.9
Knee most affected				
Right	8	7	8	6
Left	6	6	4	8
Bilateral	7	8	9	7

**Clinical** ** characteristics**				

KOA mean aduration(yr)	9.2(7.5)	11.1(8.0)	9.9(10.6)	8.8(9.3)
Hypertension mean duration (yr)	7.0(5.6)	5.8(6.7)	6.1(5.3)	6.5(5.8)
	N = 5	N = 6	N = 6	N = 5
Mean SBP(mm Hg)	132(14.7)	136(19.1)	136(17.8)	138(16.9)
Mean DBP(mm Hg)	84(19.4)	88(18.9)	87(18.5)	86(17.0)
Mean Weight(kg)	71(18.2)	74(23.1)	73(17.5)	72(19.4)
Cardiovascular history	NAD	NAD	NAD	NAD
Anti-hypertensive medication	Nifedipine	Nifedipine	Nifedipine	Nifedipine
Mean BMI	28(9.2)	27(10.1)	27(10.3)	28(8.9)

N.B:

TRT = Treatment

GRP A = Placebo Control

GRP B = Naproxen

GRP C = *Garcinia kola*

GRP D = Celebrex

### Clinical outcome

**Figure 2:**
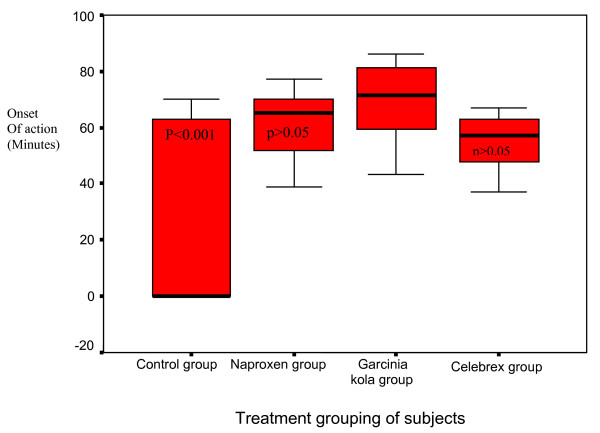
**The mean time of onset of action of the study medication (minutes)**. The mean time (minutes) of onset of symptomatic pain relief was naproxen (61.38 +/- 11.38); *G. kola *(69.13 +/- 13.12) and Celebrex (55.81 +/- 8.88). The onset of *G. kola *symptomatic pain relief was faster as compared to the control (p < 0.001) and the active comparators (p > 0.05) is shown in Figure 2.

Analysis was restricted to eighty-four patients with adequate allocation concealment. All the patients that received at least a dose of the study medications had adequate documentation of their data in each subgroup. The change in the mean WOMAC pain VAS after six weeks of *G. kola *was significantly reduced compared to the placebo (p < 0.001, CI:-2.01_-1.15, R^2 ^= 0.8). Multiple comparisons of the mean of pain change in the *G. kola *group was not lowered significantly against the naproxen and celebrex groups (p > 0.05, CI:-0.56–0.85). There was no statistically significance between the change of mean VAS pain reduction of the *G. kola*, naproxen and celebrex groups. The mean time (minutes) of onset of symptomatic pain relief were 61.4 +/- 11.3(range: 39.0–77.2); 69.1 +/- 13.1 (range:43.2–86.3) and 55.8 +/- 8.9 (range:37.1–67.0) for the naproxen,*G. kola *and celebrex subgroup respectively(p > 0.05). The onset of *G. kola *symptomatic pain relief was faster than the placebo (p < 0.001, CI: 10.0–19.9, R^2 ^= 0.87). However, *G. kola *onset of action appeared to be slower than the active comparators (p > 0.05, CI:-2.4- 9.3) as shown in Figure 2.

**Figure 3:**
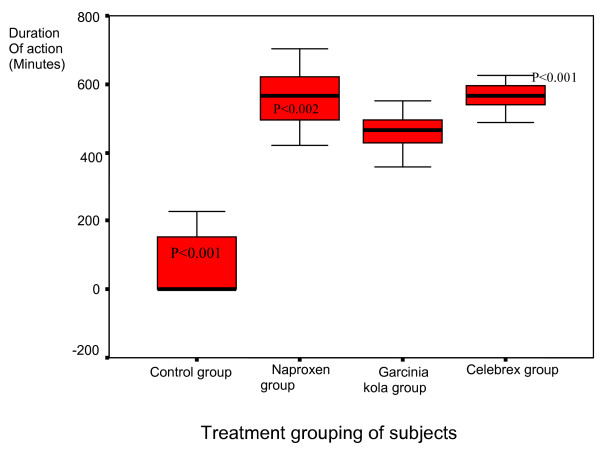
**The mean duration of action of the study medication (minutes)**. The mean (minutes) duration of therapeutic effect of study medications were naproxen (527.28 +/- 60.01), *G. kola *(454.09 +/- 55.49) and Celebrex (563.47 +/- 38.21) subgroup. The 2-way ANOVA with post hoc comparison of the mean duration of *G. kola *therapeutic action showed it was less than that of naproxen (p < 0.002) and Celebrex (p < 0.001). It was longer than the placebo (p < 0.001) as illustrated in Figure 3.

The mean (minutes) duration of therapeutic effect of study medications were 527.28 +/- 60.01 (range: 418.0–602.0), 454.09 +/- 55.49 (range: 428.8–479.3) and 563.5 +/- 38.21 (range: 546.08–580.87) for the naproxen, *G. kola *and celebrex subgroup respectively. The 2-way ANOVA with post hoc comparison of the mean duration of *G. kola *therapeutic action showed it was less than that of naproxen (p < 0.002, CI : -41.5_ -7.3) and celebrex (p < 0.001, CI: -53.6_ -19.3, R^2 ^= 0.9). This is illustrated in Figure 3.

The duration of therapeutic effect of *Garcinia kola *was longer than the placebo (p < 0.001, CI: 110.2–146.8). *G. kola *period of effect was less than naproxen and celebrex (p < 0.001, CI:-54.6_-18.1, R^2 ^= 0.972). *G. kola *subjects had improved functional mean change mobility/walking after six weeks better than the control group(p < 0.001, CI:0.3–0.5, R^2 ^= 0.8). The mean change in mobility of the *G. kola *group when compared to the active comparators was not significantly better (p < 0.05, CI:-0.15–0.15). After six weeks of study, the *Garcinia kola *subjects had significant improvement in the mean change of knee joint stiffness (p < 0.001, CI:-2.5_-1.6, R^2 ^= 0.90) and a change in mean WOMAC score (p < 0.001, CI:-26.8_-22.2, R^2 ^= 0.97) when compared to the placebo. Patients on *G. kola *reported increased walking distance 80.9 %(p < 0.001), joint stiffness relaxation, 95.2 %(p < 0.001), and improved physical function 76.2 %,(p < 0.001) compared to the placebo.

After cessation of *G. kola *use, eight (38.1%) patients were lost to follow up at clinic due to farming/trading activities which took them out of the study environment. The knee joint pain relief reported by eleven (52.4%) patients that were seen at clinic lasted for a mean period of 17.3 +/- 5.2 days (range: 9.0–26.0). There was no deterioration of the disease symptoms. The two (9.52%) hypertensive subjects (a female senior lecturer and a male principal nursing staff) who had an episode of dizziness and light headedness were excluded from the mid term follow up report. The pain relief pattern post cessation of G kola is associated with the duration of therapeutic effects (p < 0.006) as illustrated in Figure 3.

No patient in any of the four subgroup experienced symptoms suggestive of hepatic failure, hepatic dysfunction or renal failure. None had aminotransferase levels ≥ twice the upper limit of the reference range or serum creatinine levels ≥ 1.5 times the upper limit of the reference range. No statistically significant differences were observed between the groups A, B, C, and D in the proportion of patients who reported at least one or more adverse events.

Among adverse events considered to be drug related reported by about 1% of patients was peripheral edema 1(4.7%) in the celebrex group as compared to the placebo group. Two patients (9.5%) in the naproxen group had an event that was considered serious and related to the study drug: diarrhea. The side effects of *G. kola *included increased libido 9(42.8%), prolonged sleeping period 11 (52.4%) and weight loss 17 (80.9%). There was no significant cardiovascular, renal or drug induced adverse reaction to *Garcinia kola *as depicted in Table 2. No adverse event reported in the placebo group was considered both serious and related to the study medication.

**Table 2:** Patient's outcome analysis at sixth week.

**GROUP**	**Excellent**	**Good**	**Fair**	**Poor**
GROUP A			5(23.8%)	16(76.2%)
GROUP B	5(23.8%)	12(57.1%)	3(14.3%)	1(4.8%)
GROUP C	11(52.4%)	6(28.6%)	2(9.5%)	2(9.5%)
GROUP D	9 (42.8%)	10(47.6%)	1(4.8%)	1(4.8%)

## Discussion

It is now becoming increasingly clear that the development of all types of osteoarthritis involves multiple etiological factors. Meniscus injuries, misaligned fractures and post-traumatic articular cartilage surface defects are important causes of premature, localized osteoarthritis of the knee. The risk of developing posttraumatic knee osteoarthritis is increased more than threefold following major knee injury [31]. The local biomechanical risk factors which determine the site and severity of the KOA include injury, obesity, anatomical deformity and muscle weakness [32].

Guidelines from the European League against Rheumatism (EULAR) state that both pharmacological and non-pharmacological interventions are needed for optimal treatment of knee osteoarthritis [33]. The various potentially effective pharmacological interventions at the clinicians' disposal [33] highlight the need for information regarding treatment efficacy. The recent introduction of coxibs seemed to promise a reduction in serious adverse events related to NSAIDs, but this remains controversial [33]. As the evidence on the role of dietary factors in rheumatic disorders grows, it becomes increasingly important for clinicians and investigators in the field of rheumatology to familiarize them with the relevant data and appropriately apply them to clinical and public health practice.

The efficacy of *G. kola*, naproxen and celebrex was apparent for the KOA patients within the six weeks of therapy. *G. kola*'s onset of action was relatively fast with better improvement when compared with the placebo. The *Garcinia kola *positive analgesic/anti-inflammatory effect [8,9] were significant in KOA patients. This may be a useful alternative in patients with osteoarthritis who have not responded to first-line treatment with acetaminophen and in whom non steroidal anti-inflammatory drugs are contraindicated, ineffective, or poorly tolerated. As serious adverse effects are associated with oral NSAIDs, only limited use can be recommended [13].

*G. kola *is known to contain high content of bioflavonoid compounds [17] with a general anecdotal effect in folk medicine in Africa [15]. Active oxygen and free radicals are related to various physiological and pathological events, such as inflammation [34]. There is always a relationship between oxidation, infections, inflammatory reactions, and biological membrane of cells [18]. It has been reported to prevent accumulation of lipid per oxidation products and protect biomembranes against oxidative damage by acting as antioxidant [14]. It also acts as scavenger of free radicals and reactive oxygen species [19] which are not treated by traditional NSAIDs drugs or selective COX-2 inhibitors. When free radicals and reactive oxygen species accumulate in the joint could trigger additional inflammatory processes in KOA. The scavenging activity of flavonoids of *G. kola *seeds on super oxide anion radicals (O_2_) generated non-enzymically was comparable with butylated hydroxytoluene [15]. The reducing power shows that flavonoids of *G. kola *seeds are electron donors and could react with free radicals to convert them to stable products thereby terminating radical chain reaction [35] involved in knee osteoarthritis inflammatory process.

*Garcinia kola *may be acting as antioxidant to either inhibit or slow down the progression of symptomatic knee osteoarthritis. It could also act as a scavenger to remove the particles that have been observed on the surfaces of human articular cartilage following trauma and osteoarthritis [35]. The particles contained calcium and phosphorus which were identified only in structurally abnormal cartilage [35]. Bitter kola has been known to protect against the oxidation of lipoprotein, presumably through the mechanisms involving metal chelating and antioxidant activity [19,36]. The relief of pain experienced by subjects on *G. kola *could be associated with either removal of the free radicals and or revascularization of the subchondria bone through the anti-atherogenic effect. This pathway is not clear at this stage of the study. It may be through activation of the cytokines selective inhibition of inducible nitric oxide synthase which has been shown to reduce the progression of experimental osteoarthritis *in vivo *[37].

The bitter kola is believed to have aphrodisiac properties [15] probably related to its vasodilator effects on the genitalia smooth muscles. Reduction of intraosseous/subchondria pressures could be the other pathway for the reduction of knee pain experienced by patients on *G. kola*. The ability to lower intraocular pressure was earlier noted in glaucoma patients. The preliminary crude observation was confirmed scientifically in animals and human glaucoma's patients [38]. The vasodilatation induced could improve the subchondria blood circulation in knee osteoarthritis. The *G. kola *extract has been shown to have antithrombotic activities [20]. The effect of *G. kola *on chondrocyte nutrition is not clearly elucidated at present. This will form the fulcrum of future studies.

## Conclusion

*Garcinia kola *clinically appeared to have a significant analgesic/anti-inflammatory effects in knee osteoarthritis patients. *G. kola *is a potential osteoarthritis disease modifier. This study shows that *G. kola *is effective in improving locomotors function and significant pain reduction in patients with knee OA. Further study is required for standardization of dosages of *G. kola *in KOA.

## Authors' contributions

AOO conceived of the study, participated in its design and coordination and manuscript writing. ASA actively involved in the study design and coordination and manuscript writing. TOI characterized the chemistry of G Kola and participated in study coordination. OCO participated in the design and study coordination. OAO involved in the design and study coordination. EOI was involved in the study design and coordination. All authors read and approved the final manuscript.
